# Environmental Association Analyses in Plants and the Influence of Methodological Decisions

**DOI:** 10.1111/mec.70508

**Published:** 2026-08-13

**Authors:** James A. Bedford, Mark A. Carine, Mark A. Chapman

**Affiliations:** ^1^ Biological Sciences University of Southampton Southampton UK; ^2^ Life Sciences The Natural History Museum London UK

**Keywords:** environmental association analysis, GWAS, landscape genomics, rice

## Abstract

Climate change is predicted to impact existing ecological systems, leading to a requirement to improve our understanding of environmental adaptation in plants, particularly for conservation and the development of environmentally tolerant crop varieties. Environmental association analyses (EAAs) are a group of landscape genomic methods for detecting genetic markers or genes associated with environmental factors and therefore provide a top‐down approach of studying the genetics of environmental adaptation. Here we review 138 EAA studies in plants, revealing the large diversity of methods at each stage of the analysis, including marker filtering, EAA model selection, the significance criteria used, candidate gene selection and any downstream analyses conducted. Following this, we compared four frequently used EAA models using genetic data from a panel of traditional rice ( *Oryza sativa* ) varieties from Vietnam, revealing a greater proportion of significant markers for recent GWAS models (FarmCPU and BLINK) compared to single locus mixed linear models (MLM) and latent factor mixed models (LFMM). Between zero and 22 environmentally associated markers overlapped between models and several genes in proximity to these loci have putative links to abiotic factors. Overall, this highlights the challenges in comparing the results of EAA studies, particularly regarding applying the results to crop breeding for future climates.

## 1 Introduction

Global climate change is predicted to drive an increase in day and night temperatures, heat wave events, heavy precipitation and droughts (Wassmann et al. 2009). Climate modelling under the Representative Concentration Pathway (RCP) 2.6 scenario, which would entail a large reduction in greenhouse gas emissions, is predicted to result in an increase in global temperature of approximately 2°C by 2100. An intermediate scenario (RCP 4.5) is expected to exceed 2°C, with an upper likely range of 2.6°C (IPCC 2014). Even under RCP 2.6 there will be considerable change to existing ecological systems. Thirty three percent of land is predicted to experience a significant change in their ecologies and a shift in the types of plants supported in these regions, with this approximately doubling to 68% of land under the RCP 4.5 scenario (Conradi et al. 2024). For example, wet tropic regions are projected to experience higher temperatures and reduced precipitation, leading to an increased favourability towards drought adapted species (Conradi et al. 2024). In general, a trend of range shifts towards the poles and to higher elevations are expected (Parmesan and Hanley 2015). There may be benefits of elevated CO_2_ and a prolonged growing season associated with a shift in climate, particularly for C_4_ grasses such as wheat and rice, however this comes at the cost of increased water demand and poses a challenge as the frequency of extreme heat and drought events are likely to increase (Rezaei et al. 2023). It is therefore increasingly important to understand how plants adapt to their environments to inform conservation efforts, e.g., to identify appropriate climate‐tolerant genotypes and environments for planting, or to detect alleles adapted to local environmental conditions that could inform crop breeding. For instance, a gene conferring adaptation to dry climates may be beneficial for drought tolerance going forward.

Environmental association analyses (EAAs), also referred to as genome‐environment association (GEA) analyses, form a branch of landscape genomics that functions by statistically relating genetic markers with environmental conditions often using models that account for population structure (Rellstab et al. 2015). Loci associated with environmental conditions can then be investigated, revealing candidate genes that may be involved in environmental adaptation across the population analysed. Following identification of environmentally associated loci, marker validation can be used to confirm associations, accounting for the false positive rates of genome‐wide analyses. For instance, reciprocal transplant experiments using candidate SNPs to predict fitness changes can provide strong support for the presence of local adaptation (Rellstab et al. 2015).

Despite the potential uses of these approaches, there are no ‘gold‐standard’ pipelines for a successful EAA and there is no apparent standardisation across the field. A large array of methodological options for the statistical models used, type of genetic marker, marker filtering criteria, significance criteria, multiple test correction and follow up analyses are employed across studies. A limited number of studies have attempted to address this issue by providing guidance on the different stages of the EAA process Rellstab et al. (2015), a key review in this field, provides an overview of a typical EAA workflow, including the options for sample design, sources and processing of environmental data and a summary of each of the frequently used models. This includes regression, matrix correlations, general linear models (GLM), mixed linear models (MLM), latent factor mixed models (LFMM) and GWAS approaches. However, in the 10 years since this review, several new GWAS methods have been published that have greater power and computational efficiency, such as FarmCPU (fixed and random model circulating probability unification; Liu et al. 2016) and BLINK (Bayesian‐information and linkage disequilibrium iteratively nested keyway; Huang et al. 2018).

Ahrens et al. (2018) assessed the methods of 66 studies that applied both EAA and outlier analyses across a variety of plant, animal and insect species, comparing factors such as the number of markers, environmental variables and individuals and the reporting of parameters including minor allele frequency (MAF), population structure and linkage disequilibrium. They found that the genomic sampling effort, i.e., the number of genetic markers, had a greater influence on the detection of significant associations than the biological sampling effort and that the level of detail in reporting results varied considerably across studies. Previous studies have also simulated changes to MAF and missing data and the effect on rates of true and false positive associations when using BAYENV2 and LFMM approaches, finding that in general, relaxed thresholds result in more true positives at the expense of more false positives (Ahrens et al. 2021). Others have focused on defining best practices for data curation, reporting and deposition to enable meta‐analyses of EAA studies in the future (Waldvogel et al. 2020).

Collectively, these studies have demonstrated that selection of parameters can vary across and impact the results of EAAs. We aim to expand on these findings by focusing specifically on plant species, including recent EAA methods, comparing downstream analyses and the applications of the research. To this end, literature searches were conducted to identify EAA studies in plants, followed by an analysis of their methods with the intention of characterising these studies, identifying frequently used approaches and critiquing their proposed applications.

We then apply four common EAA models to a panel of traditional rice ( *Oryza sativa* L.) varieties from Vietnam. Rice is a globally important crop species with Vietnam positioned as the fifth largest producer and third largest exporter in 2017 (Maitah et al. 2020) and includes highly productive regions such as the Red River and Mekong Deltas and has notable environmental gradients across the country, especially North–South. Traditional landrace varieties are locally cultivated and are therefore more likely to be locally adapted to such environmental conditions, making this panel suitable to apply to EAA models. The outputs of these models were compared, highlighting markers and genes commonly detected between methods to provide insight into the extent to which different methods resolve the same associations, while also producing a list of candidate putative locally adaptive genes that could be applied to rice breeding.

## 2 Methods

### 2.1 Identification of Previous Plant EAA Studies

EAA studies were identified by literature searches in June 2026 across Web of Science and Google Scholar, limiting the search to studies published prior to 2026 using combinations of the search terms ‘Environmental association analysis’, ‘Genome‐environmental association analysis’, ‘landscape genomics association’ and ‘plants’. From each study the following information was obtained where available: Year, species analysed, number of accessions/individuals analysed, EAA statistical method used, type of genetic marker analysed, marker filtering criteria applied (pre‐outlier analysis, MAF and missing data), significance thresholds, number of markers and significant markers, the number of associated genes and whether a GO‐enrichment analysis was conducted (Table S1). From this information, we determined the distribution of markers as random or non‐random and whether markers were pre‐filtered using outlier analyses (e.g., *F*_ST_). Markers were determined to have a non‐random distribution if they were identified through targeted sequencing, e.g., of preselected candidate genes. The proportion of significant environmentally associated markers was also calculated. For studies analysing crop species, the literature citing them was additionally scanned to identify any follow up experiments, for example, using the results to inform crop breeding or if transgenics were used to validate candidate markers or genes.

### 2.2 Statistical Analysis of EAA Studies

The number of genetic markers analysed across studies was compared using Kruskal–Wallis based on whether markers showed a targeted vs. non‐targeted distribution across the genome or whether markers were pre‐filtered using outlier analyses (e.g., *F*_ST_) vs. no pre‐filtering. For studies using a random distribution of SNPs and no pre‐outlier detection (i.e., genome‐wide SNPs), the effect of statistical method on the proportion of significant environmentally associated SNPs was analysed using Kruskal‐Wallis. For the same set of studies, Spearman's rank correlation analysis was used to test for correlations between the SNP filtering criteria (MAF and missing data) and the proportion of significant SNPs.

### 2.3 *Oryza sativa* Traditional Variety Panel

To investigate the effect of EAA method on the detection of environmentally associated SNPs, we used SNP data from genotype‐by‐sequencing of a panel of *O. sativa* accessions (Phung et al. 2014). This dataset consists of 182 Vietnamese varieties genotyped and imputed across 21,814 markers with location data at the province level. Therefore, the location of each accession was set to the centre of their province of origin. A subset of the panel was improved varieties, which were removed from the analysis, keeping only traditional varieties that are most likely to be landraces and expected to display local adaptation. This reduced the size of the panel to 163 accessions and after recalculating MAF using a threshold of 5%, 21,416 markers remained.

Climate data were obtained from the WorldClim database version 1.4 (Hijmans et al. 2005) using the R package *raster* (Hijmans et al. 2020) in RStudio v4.2.2. The WorldClim data set contains global climate information interpolated from observed data collected between approximately 1960 and 1990. Nineteen bioclimatic variables (9 temperature‐associated, 6 precipitation‐associated variables, and 4 associated with both temperature and precipitation; Table S2) were retrieved using the centre point of each province to download specific grid values. The largest grid size ca. 18 km^2^ grids (10 arc‐min resolution grid) was used due to the absence of specific coordinates.

Composite climate variables, principal component 1 (PC1), PC2 and PC3, were derived from the climatic data via principal component analysis (PCA) on all climate variables using the RStudio core function ‘princomp’ with parameters cor and scores enabled. Correlations between variables at each sample location were analysed with Spearman's rank correlation and based on the correlation data, groups of highly correlated variables (*ρ* ≤ −0.8; *ρ* ≥ 0.8) were reduced to a single variable to limit the effects of multiple testing. After this, the following variables were analysed: BIO1, BIO2, BIO3, BIO5, BIO8, BIO12, BIO14, BIO15, BIO18, BIO19 and PC1‐3.

### 2.4 EAA

Four of the frequently used EAA models were run, comparing their ability to detect environmentally associated (EA) markers: MLM (Yu et al. 2006), FarmCPU (Liu et al. 2016) and BLINK (Huang et al. 2018) implemented in GAPIT3 (Wang and Zhang 2021) and LFMM (Frichot et al. 2013) implemented in R v4.2.2 using RStudio and the package *lfmm* (Caye et al. 2021). For the GAPIT models, population structure was incorporated using the first two PCs of the genomic data, with MLM and FarmCPU additionally using kinship matrices to account for relatedness between accessions. BLINK is a model that uses fixed effects, MLM uses random effects and FarmCPU is a hybrid model that uses both fixed and random effects. LFMM was implemented using a *k* = 2 model, selected using PCA, an assessment of QQ plots and previous STRUCTURE analysis (Phung et al. 2014). SNP data was converted to a numerical format as required for LFMM using the function *raw.data* in the package *snpReady* (Granato and Fritsche‐Neto 2018). Significantly EA SNPs were identified as those passing an FDR threshold of 0.05. Overlap in significant markers between models was visualised using Venny 2.1 (Oliveros 2015). Genes in close proximity (±10 kb) to significant regions were identified using the Osa1 release 7 genome browser (http://rice.uga.edu/cgi‐bin/gbrowse/rice/), to which the marker data was previously aligned, and putative functions determined through literature searches.

## 3 Results

### 3.1 EAA Studies, Taxonomic Representation and the Influence of Methodological Decisions

**FIGURE 1:**
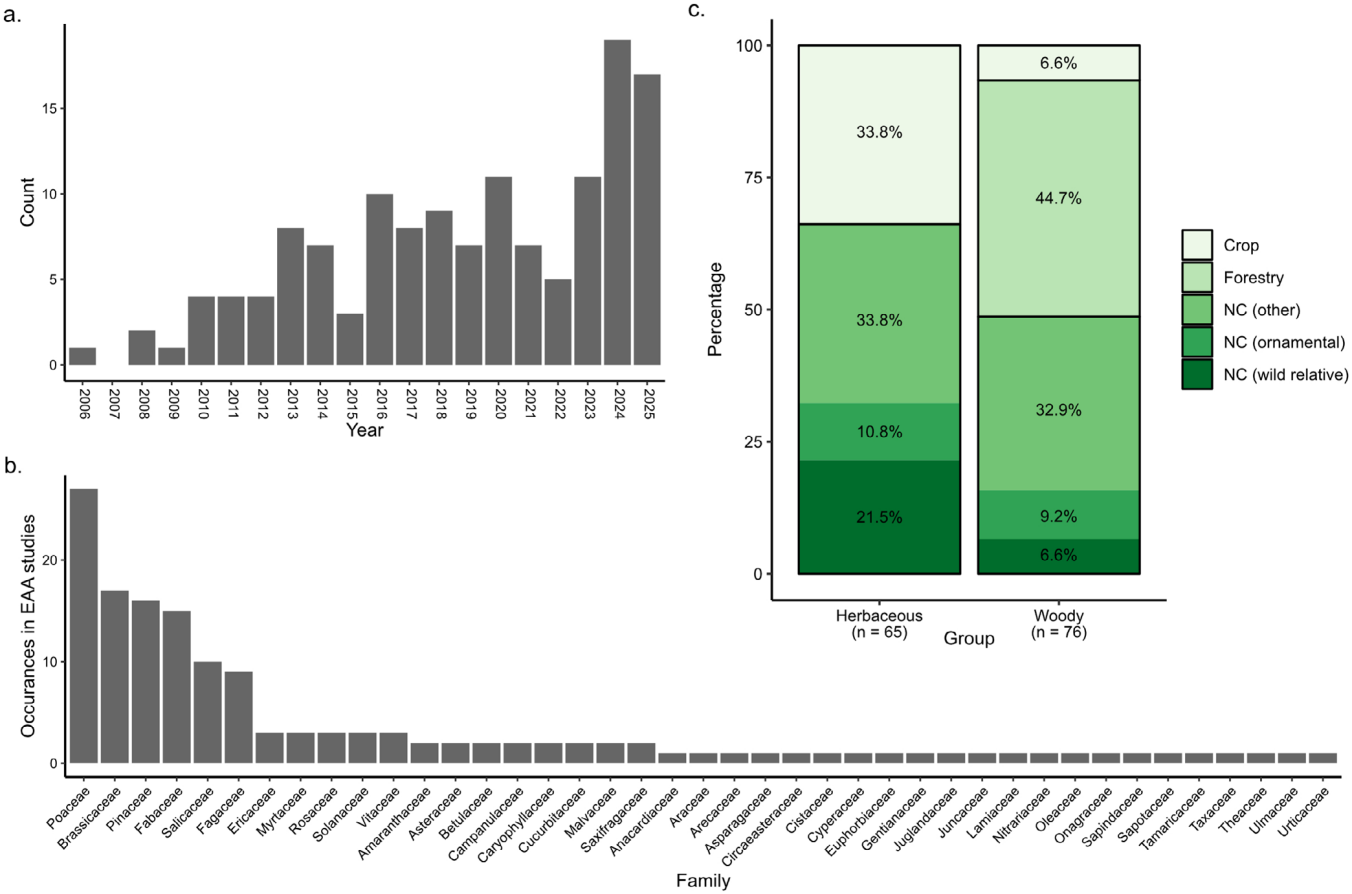
(a) Environmental association analyses (EAA) in plants from 2006 to 2025. (b) Plant families represented across studies. The total count differs from the number of published studies as several investigate multiple families. (c) Proportion of species/subspecies classed as crops, forest species, or non‐crops (NC) for herbaceous (65) and woody (76) species. The total number of species examined is 141.

From 2006 to 2025, 138 plant EAA studies were identified in the literature search and there were between zero (in 2007) and 19 (2024) publications per year. There were fewer publications between 2020 and 2023, potentially coinciding with restrictions on laboratory and field work during this period of global pandemic (Figure 1a). Publication of EAA studies appears to be increasing again, with the most publications per year in 2024 and 2025. Across these 138 studies, a total of 139 species, 77 genera and 41 families are represented, with 14 studies covering two or more species or subspecies (Table S1). Poaceae, Brassicaceae, Pinaceae, Fabaceae and Salicaceae are the top five most studied families, collectively represented in 61% of publications (Figure 1b). Angiosperms are the most studied with only two gymnosperm families present (Pinaceae and Taxaceae; Figure 1b). In total, 55% of the 139 species are trees and other woody species (e.g., shrubs and vines).

For herbaceous taxa, most species studied are non‐crop species (66%), of which one third are crop wild relatives (Figure 1c). However, for woody taxa, the proportion of species with commercial value (crops and forestry species) is greater (51%; Figure 1c).

**FIGURE 2:**
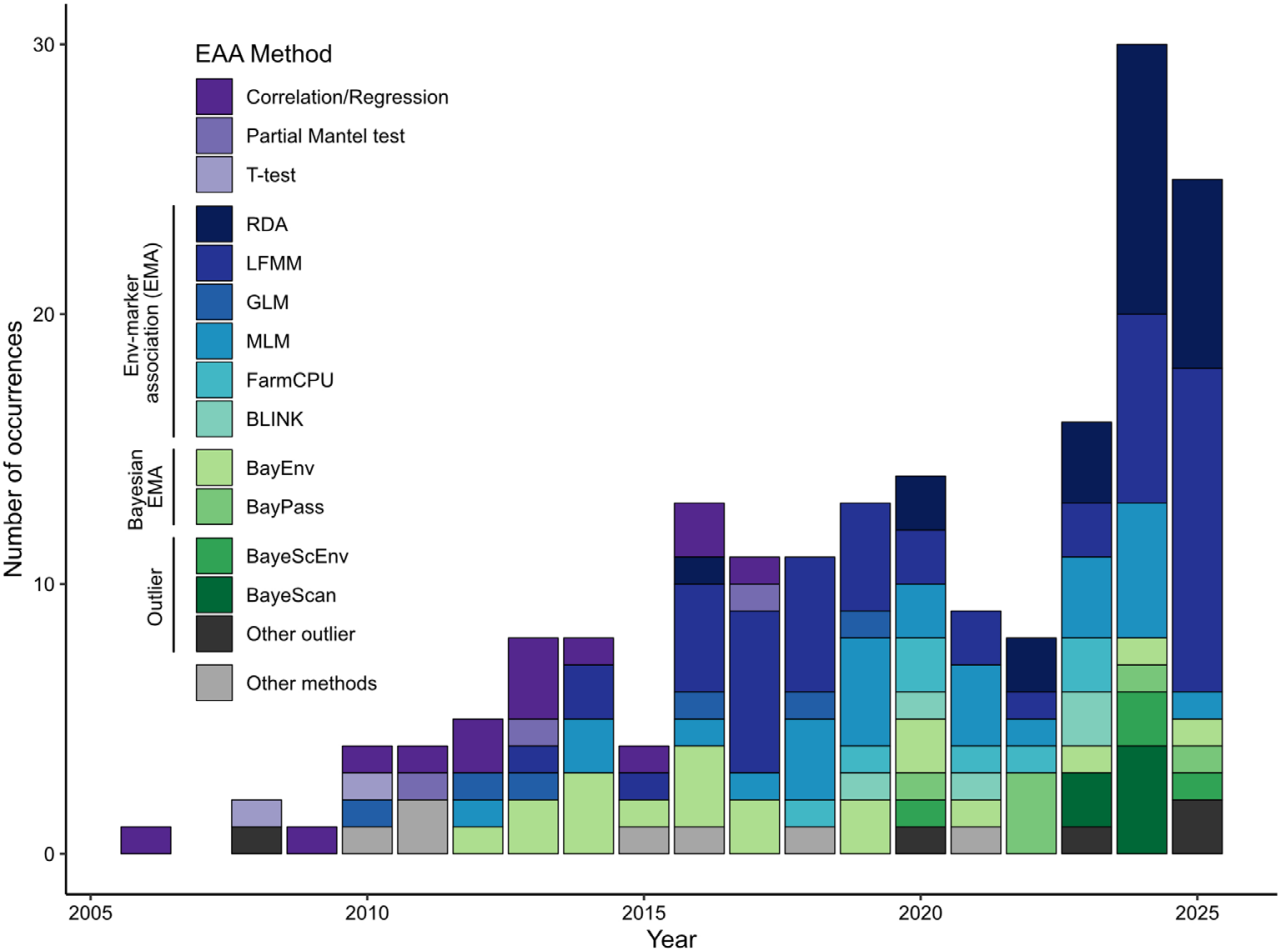
Methods used across EAA studies over time, broadly grouped into environment‐marker association (EMA) methods, Bayesian EMAs and outlier‐based methods. GLM, Generalised Linear Model; LFMM, Latent Factor Mixed Model; MLM, Mixed Linear Model; RDA, Redundancy Analysis.

LFMM, MLM, redundance analysis (RDA) and Bayenv (version 1 or 2) are the most frequently used over this period, present in 49, 27, 25 and 20 studies respectively, with 40 of the 138 studies (29%) using multiple methods to detect genetic‐environment associations. Other methods include spatial analyses, generalised linear models, partial Mantel tests, *t*‐tests, other Bayesian approaches, and recent variations of GWAS implementation (e.g., BLINK and FarmCPU). Regression/correlation analyses are more frequently used in earlier studies, whereas GWAS and multivariate (e.g., RDA) methods have gained popularity in recent years (Figure 2).

**FIGURE 3:**
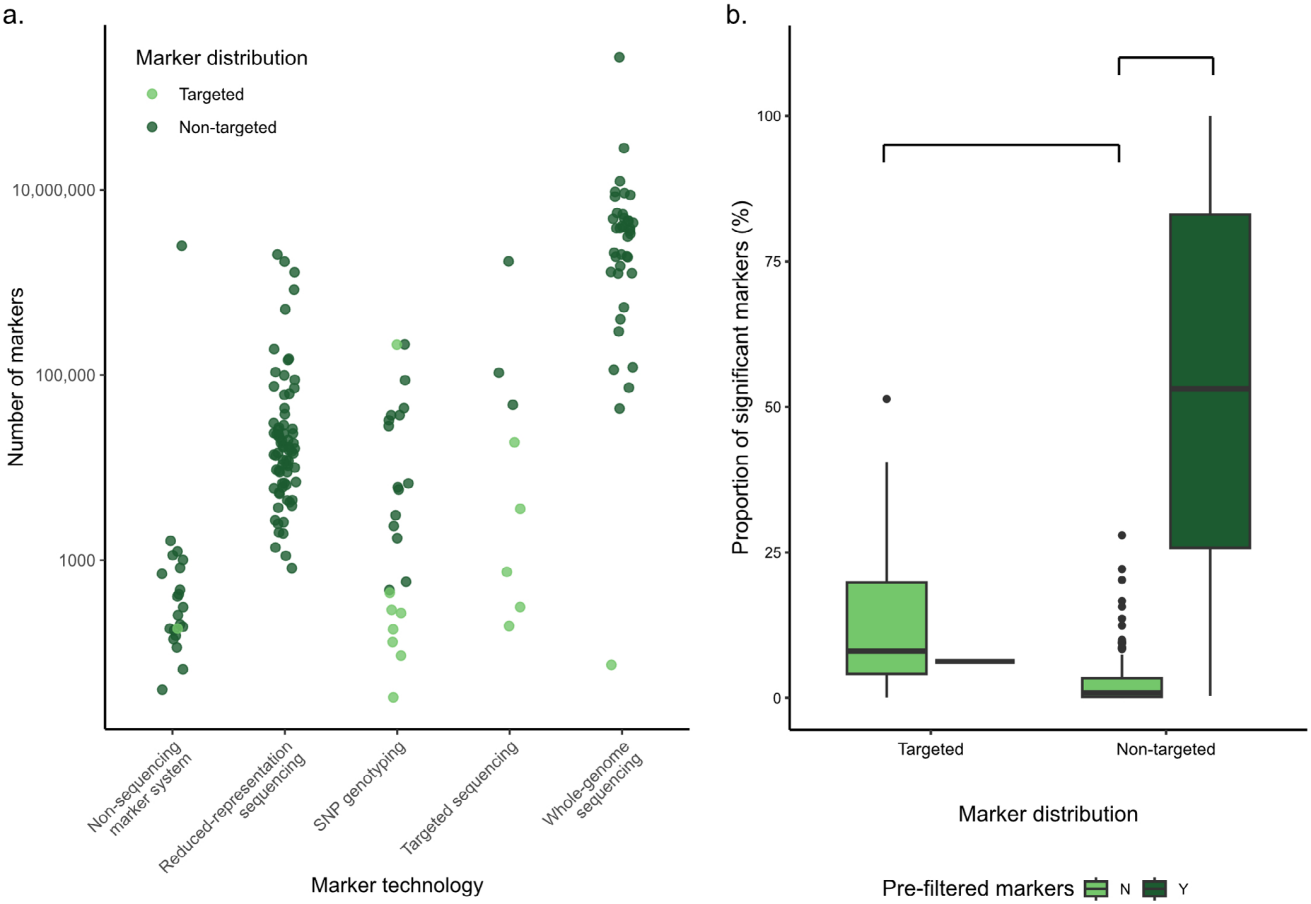
(a) Total marker counts across all marker datasets used in the 138 studies, grouped by marker technology and separated by whether marker selection was random or targeted to specific genes/markers. (b) Percentage of markers that were significantly environmentally associated, separated by marker distribution and whether pre‐filtering (e.g., *F*_ST_) was used. Significant differences are highlighted above the plot.

SNP markers are analysed in ~85% of studies, although their use only began in 2010, prior to which amplified fragment length polymorphism (AFLP) markers were frequently analysed. Marker distribution takes two forms, non‐targeted (e.g., SNP array, reduced‐representation sequencing, and whole‐genome sequencing [WGS]) or targeted, where sequencing of only candidate genes occurs. Alberto et al. (2013), for example, carried out targeted sequencing of 106 candidate genes for bud burst timing in sessile oak ( *Quercus petraea* ) that displays clinal patterns in common garden experiments. Keller et al. (2017) limited the association analysis to 27 flowering time genes in Balsam poplar ( *Populus balsamifera* ) and Rodriguez et al. (2016) analysed 131 SNPs across 100 genes in wild common bean accessions ( *Phaseolus vulgaris* ) that were ‘putatively involved in adaptation to both biotic and abiotic stresses’ based on previous studies. As expected, on average the number of markers analysed is greater in studies using whole‐genome sequencing, compared to reduced‐representation sequencing (e.g., genotyping by sequencing [GBS] and restriction‐site associated DNA sequencing [RAD‐seq]), SNP genotyping (e.g., SNP arrays), targeted sequencing (e.g., target enrichment and exome capture) and non‐sequencing marker systems (e.g., AFLPs and start codon targeted [ScoT] polymorphisms; Figure 3a). For marker selection, there is an additional split in the experimental design where either (i) candidate locally adaptive genetic markers are pre‐filtered based on outlier analyses such as *F*_ST_, to select markers that display significant differentiation between populations, or (ii) no outlier detection occurs and the whole marker data is analysed. Pre‐filtering based on outlier analyses is less frequently used, with 16 publications using this method, an example of which is Richardson et al. (2009) that investigated *F*_ST_ of 66 AFLP markers across 15 populations of silver pine ( *Pinus monticola* ), identifying eight outliers, four of which were significantly correlated with bioclimatic variables.

Where the number of significant markers was reported (199 analyses across 130 studies), whether or not markers were selected based on pre‐filtering [e.g., *F*_ST_] and/or whether markers were or were not targeted significantly affects the proportion of environmentally associated (EA) markers identified (Kruskal‐Wallis: χ^2^ = 49.454; df = 3; *p* < 0.001). As expected, when no pre‐filtering is used, the proportion of EA markers is significantly greater when using a targeted selection of markers compared to non‐targeted markers (Figure 3b). When non‐targeted markers are used, pre‐filtering results in detection of a significantly greater proportion of EA markers (Figure 3b).

**FIGURE 4:**
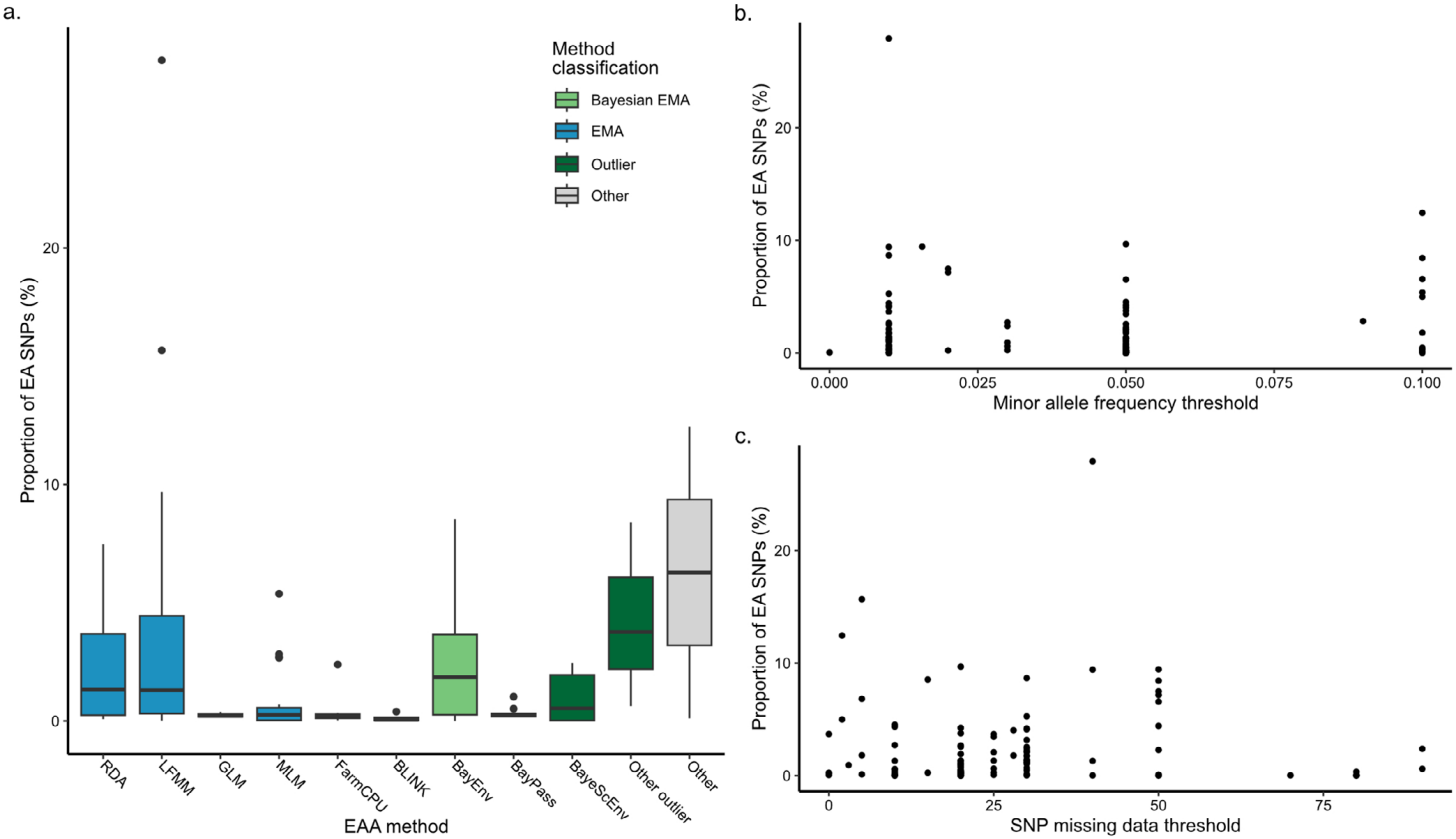
Analyses where no pre‐filtering of candidate markers and a non‐targeted distribution of SNPs are used. Graphs show the proportion of significant environmentally associated (EA) SNPs (a) across methods, where the method had been used in more than one study, (b) compared to the minor allele frequency and (c) compared to the SNP missing data thresholds. EMA, Environment‐Marker association.

Differences in how SNPs are filtered, the significance thresholds used, and statistical approach selected vary among studies and influence detection of EA SNPs. Filtering of SNPs based on minor allele frequencies (MAF) and missing SNP data across all individuals in the accession panel are common practices when analysing large SNP datasets, the aim of which is to reduce the likelihood of false positives arising from rare variants (Ahrens et al. 2021). The most frequently used statistical method in studies using a random distribution of SNPs and no pre‐outlier detection (*n* = 161 analyses) is LFMM (48), MLM (25), RDA (24), Bayenv (13) and BayPass (13). For the subset of methods used more than once, and where the proportion of significant SNPs could be calculated per model, there was a significant effect of EAA method on the proportion of significant EA SNPs detected (Kruskal‐Wallis: χ^2^ = 32.516; df = 14; *p* = 0.003; Figure 4a), although the effect is small as no specific differences were identified post hoc.

Across the 138 EAA studies, filtering by MAF and missing data is reported for 94 and 84 studies, respectively. A threshold of 5% was used most frequently (*n* = 51) when filtering SNPs with low MAF, but the threshold varied from 0% to 10%. The missing data threshold applied for removal of SNPs ranged from 0% to 90%, with 0% being the most stringent threshold (as no missing data is permitted) and with 20% frequently used (*n* = 26). There is no significant correlation between the MAF threshold used and the proportion of environmentally associated SNPs identified (Spearman's rank: *ρ* = 0.15; *S* = 358,060; *p* = 0.09; Figure 4b), or between the proportion of EA SNPs and the SNP missing data threshold (Spearman's rank: *ρ* = 0.10; *S* = 293,369; *p* = 0.27; Figure 4c).

In addition to the factors highlighted previously, the significance criteria used and the method of selecting candidate SNPs or genomic regions for downstream analyses also vary across studies. For example, a stringent Bonferroni threshold (0.05/number of tests) was frequently used to determine marker significance; however, other significance criteria included an adjusted *p* value threshold of 0.0001 or smaller, or an adjusted threshold using false discovery rate (FDR) (although the threshold for this varied substantially from 0.25 to 0.01). For Bayesian approaches, the significance threshold varied from requiring a Bayes Factor (BF) > 20 (stringent) to BF > 3 or selecting markers in the 99th percentile of BFs. This variation in stringency is expected to influence detection of true and false positives.

For candidate marker selection, this varies from investigating genes surrounding significant EA genomic regions (Bandillo et al. 2017), selecting the SNPs with the smallest *p* values (Yoder et al. 2014), focusing on genomic regions that are significant across multiple environmental variables (Wang et al. 2023) or investigating the overlap of candidate SNPs across multiple methods (De La Torre et al. 2019).

**FIGURE 5:**
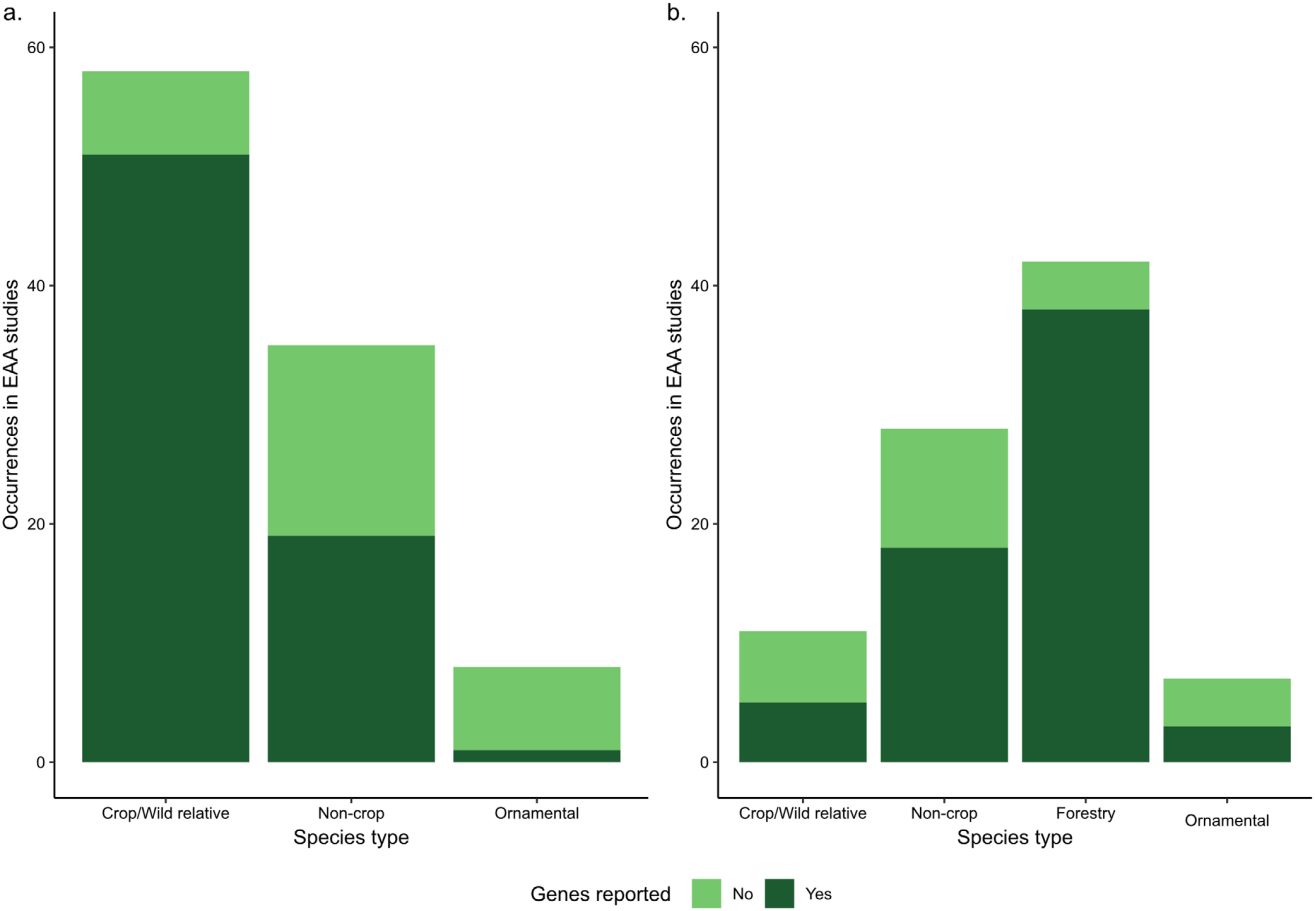
Species occurrence across 138 EAA studies and whether candidate genes are reported, grouped by species type for (a) herbaceous taxa and (b) woody taxa.

Once EA markers are identified, follow up analyses may include gene identification, gene ontology (GO) enrichment analysis to identify processes associated with genes, or generation of transgenics for validation or functional classification. This is, in theory, possible for the 108 of the 138 studies that mention specific gene names or functions, although the extent of gene identification across EAA studies varies widely from a single gene to > 3000. Where no reference genome is available, gene identification is not carried out, such as for black alder ( *Alnus glutinosa* , De Kort et al. 2014). Additionally, marker type influences gene identification: for markers with no sequence information, e.g., SCoT, AFLP, ISSR, it is challenging to identify genes and gene function (Li et al. 2017). Related to this, the proportion of studies highlighting genes is greater for those investigating crop species and crop wild relatives, compared to non‐crop species (Figure 5), potentially because crops species tend to be the focus of genome sequencing efforts.

Gene ontology (GO) enrichment analyses aim to reveal the broad functions, biological processes and localisations of gene sets by testing for enrichment of assigned GO terms compared to a background set (Primmer et al. 2013) and can be used following EAA (Rellstab et al. 2015). Where genes were identified, enriched GO terms were reported only 30% of the time, representing 32 of the 108 studies. For example, in *Medicago truncatula*, Guerrero et al. (2018) detected enrichment of terms related to mineral ion transport in a gene set identified using LFMM analysis on drought and salinity factors. Additionally, analysis of EA across 23 populations of *Pinus densata* revealed 2025 SNPs associated with temperature, water availability, soil organic carbon, UVB and altitude. These mapped to 703 genes with enrichment of GO terms related to protein transport, regulation of gene expression and regulation of metabolic processes (Zhao et al. 2020). Furthermore, across 244 accessions of wild barley ( *Hordeum vulgare* ssp. *spontaneum* ), EAAs using simple RDA and outlier (BAYPASS) approaches resulted in the detection of 159 and 125 genes, respectively, with enriched GO terms related to fatty acid metabolism, seed germination, response to cytokinins (plant hormone) and alpha amylase activity (Chang et al. 2022). Frequently, no enriched GO terms are identified (Günther et al. 2016; Jordan et al. 2017; Tabas‐Madrid et al. 2018; Chang et al. 2022; Bedford et al. 2023).

In terms of crop improvement, EAA studies have the potential to identify genetic variation arising from adaptation to climate (Rellstab et al. 2015). Validation of genetic effect may be conducted with development of transgenics, common garden experiments or validation of genetic‐environment associations in independent populations (Rellstab et al. 2015). Once confirmed, these genetic markers may be used in marker‐assisted backcrossing to develop crop varieties adapted to different environments. For example, an analysis of barley landraces identified two significant SNPs associated with the number of potential vernalization days and number of frost days, both of which mapped to the gene *CBF4*, known to be involved in cold acclimation (Contreras‐Moreira et al. 2019). Genes such as these may be beneficial targets for crop breeding. As the broad context for crop‐based EAAs is often linked to breeding (as mentioned in 35 out of 43 crop studies), we analysed these publications and all literature referencing them, aiming to identify any that used the EAA results to inform transgenics or crop breeding. Of the 43 crop studies, none directly generated transgenics or functionally tested targets to confirm the effect of SNPs or candidate genes. Additionally, scanning the publications referencing the 43 crop EAAs revealed only two examples where we considered there was evidence that the associated genes were being used in the context of breeding, and in both it was early‐stage exploratory research. These were studies where (1) environmentally associated genes from sugarcane (Yang et al. 2019) being added to a SNP‐chip for sugarcane breeding (Chen et al. 2026), and (2) environmentally associated genes from barley (Lei et al. 2019) were evaluated for environmental genomic selection in barley (Halpin‐McCormick et al. 2025). Two further examples of validation but outside a breeding context were found where environmentally associated genes in sunflower (Todesco et al. 2020; Andrew and Rieseberg 2013) were validated in the context of wild sunflower local adaptation (Todesco et al. 2022; Innes et al. 2024).

### 3.2 Method Comparison Using a Panel of Traditional *O. sativa* Varieties From Vietnam

**FIGURE 6:**
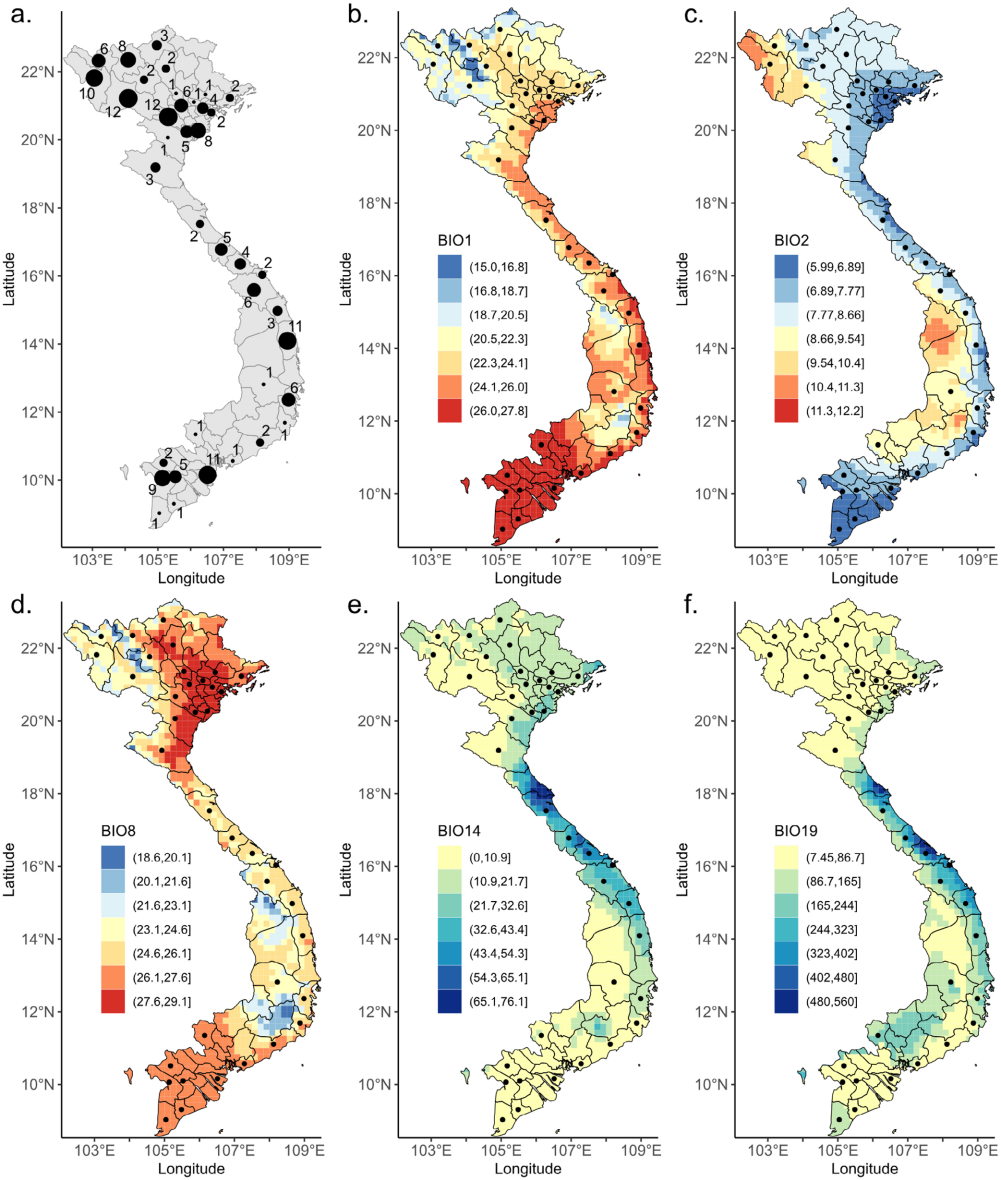
(a) Number of *O. sativa* accessions in each province of Vietnam. (b) BIO1 annual temperature (°C); (c) BIO2 mean diurnal range (°C); (d) BIO8 temperature of the wettest quarter (°C); (e) BIO14 precipitation of the driest month (mm) and (f) BIO19 precipitation of the coldest quarter (mm) across the sample region.

To further investigate the effect of EAA method on the detection of environmentally associated SNPs, we ran four different EAA models on SNP data from a panel of 163 landrace *O. sativa* from Vietnam, consisting of the two *O. sativa* subspecies, *japonica* and *indica*. The accessions are distributed across most provinces of Vietnam (Figure 6a), covering agriculturally important regions including the Red River delta in the north and the Mekong River delta in the south. It is generally cooler in the north compared to central and southern provinces and has greater precipitation in central regions (Figure 6). For example, annual temperature (BIO1) ranges from ~16°C to 27°C (Figure 6b), mean diurnal range (BIO2) varies from ca. 6°C to 10°C (Figure 6c) and the precipitation of the coldest quarter ranges from 36 mm to 533 mm, being generally greater in central regions (Figure 6f). Based on a visual assessment of the estimated accession location and climate variation across each province, using the province centre coordinates to obtain the climate data provides a reasonable prediction (Figure 6). Although potential inaccuracy of climate data may influence the detection of EA markers, the same climate data was applied to each model, so allowing the relevant comparisons to be made.

**FIGURE 7:**
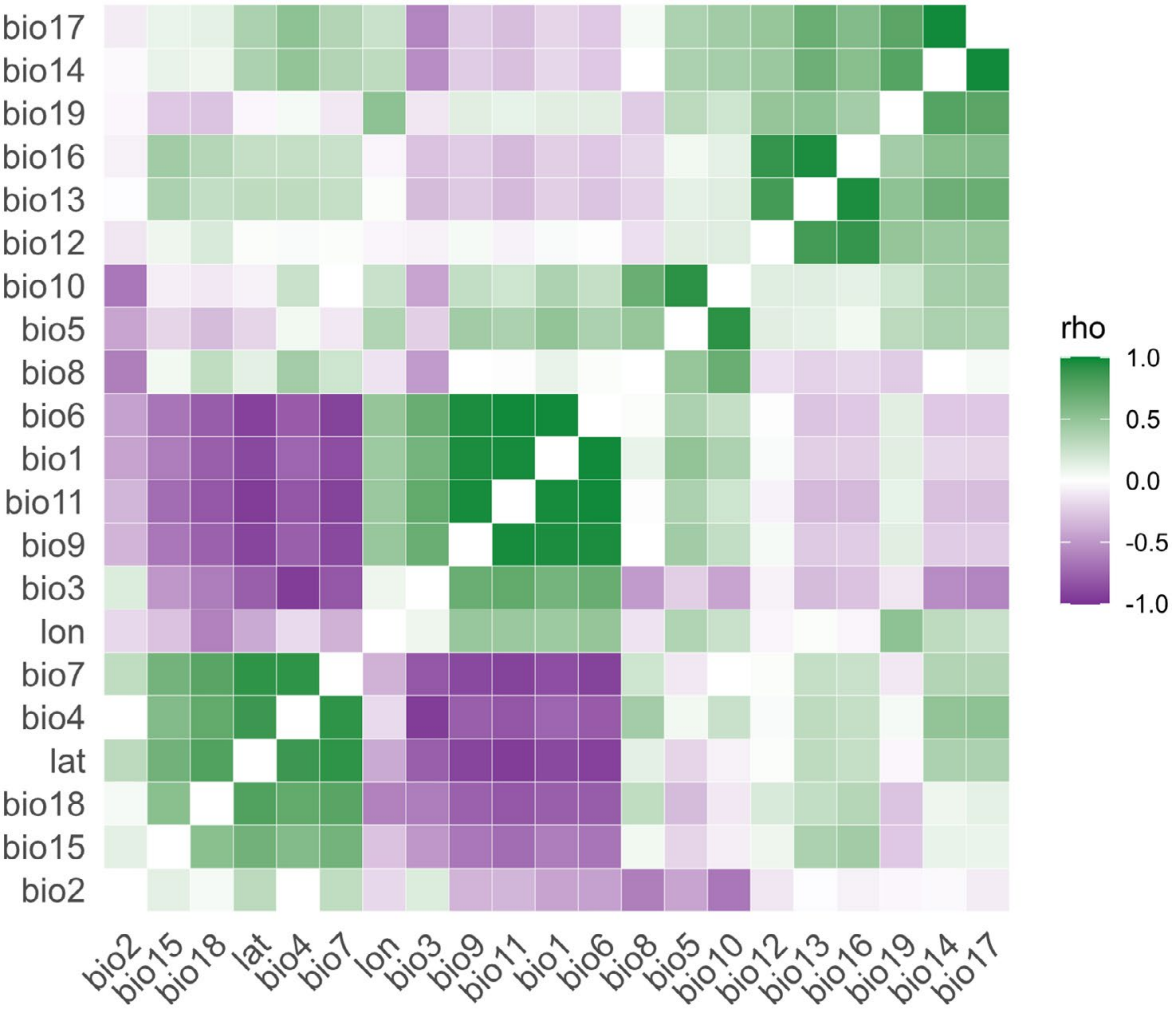
Spearman's rank correlation (*ρ*) between 19 bioclimatic variables (described in Table S2) and location coordinates (lon, longitude; lat, latitude).

A correlation analysis between climate variables revealed that, as expected, many of the temperature associated climate variables were highly correlated with each other and with latitude. Some of the precipitation variables were correlated with the other precipitation variables, although to a lesser extent than between temperature variables. BIO2, BIO8, BIO15, BIO19 and longitude were not highly correlated with any other variables (Figure 7).

**FIGURE 8:**
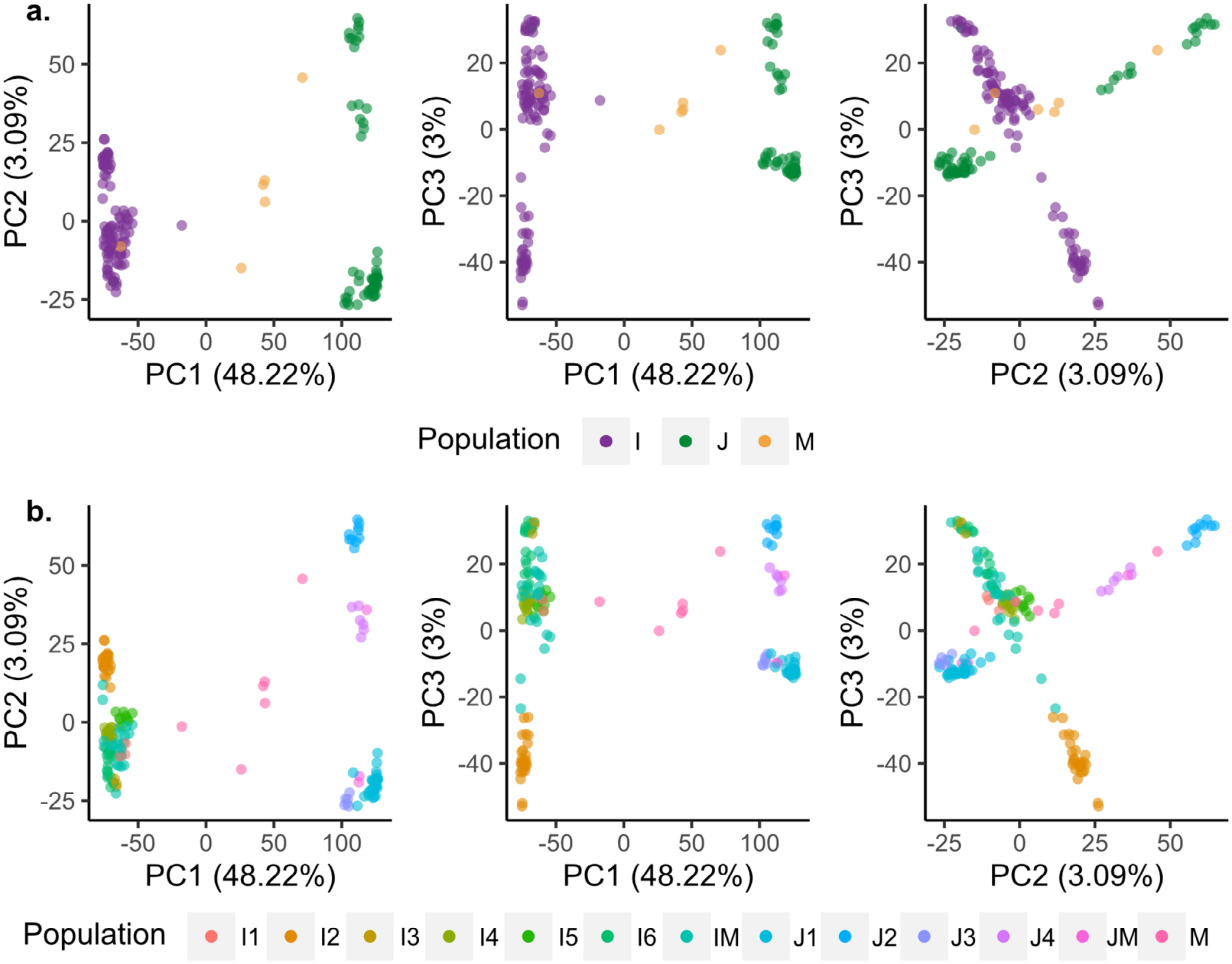
Population structure across the *O. sativa* accession panel, presented as a PCA of 21,416 markers across 163 accessions. Each dot represents a population, coloured based on designations from Phung et al. (2014) using (a) DArT markers and (b) genotype‐by‐sequencing data. The percentage variance explained by each PC axis is in parentheses. I = *O. sativa* subsp. *indica*; J = *O. sativa* subsp. *japonica*; M = admixed.

Across the panel of 163 accessions, PCA of the marker data revealed a large separation across the first principal component (PC) of 48.22% with less contribution of PC2 (3.09%) and PC3 (3%) axes (Figure 8a). Overlaying population designations from a structure analysis of 241 DArT markers (Phung et al. 2014) onto the PCA supported that PC1 corresponds to the split between *japonica* and *indica* subspecies with few admixed individuals (Figure 8a). Additionally, overlaying populations from structure analysis of 1275 SNPs, which resulted in six *indica*, four *japonica* and three admixed groups (Phung et al. 2014), again revealed the separation of *japonica* and *indica* along PC1 but with less distinct grouping for the subpopulations (Figure 8b). Therefore, the largest genetic differences within the panel are, as expected, between *indica* and *japonica* populations.

In total, 142 unique FDR significant SNPs were detected across the four EAA methods (Table S3). MLM detected three significant SNPs, i.e., 0.00014% of markers, all from one region of chromosome 8 associated with the precipitation of the coldest quarter (BIO19). LFMM detected three and two significant SNPs in chromosomes 3 and 4, respectively (0.00023%), associated with mean diurnal range (BIO2). The two other GWAS methods detected a greater proportion of significant SNPs, however, they were rarely grouped into distinct regions. BLINK detected 55 SNPs in total (0.0026%) associated with all climate variables except BIO12 and BIO15 and FarmCPU detected 103 SNPs in total (0.0048%) associated with all climate variables except BIO12. Manhattan and QQ plots are presented in Figure S1.

**FIGURE 9:**
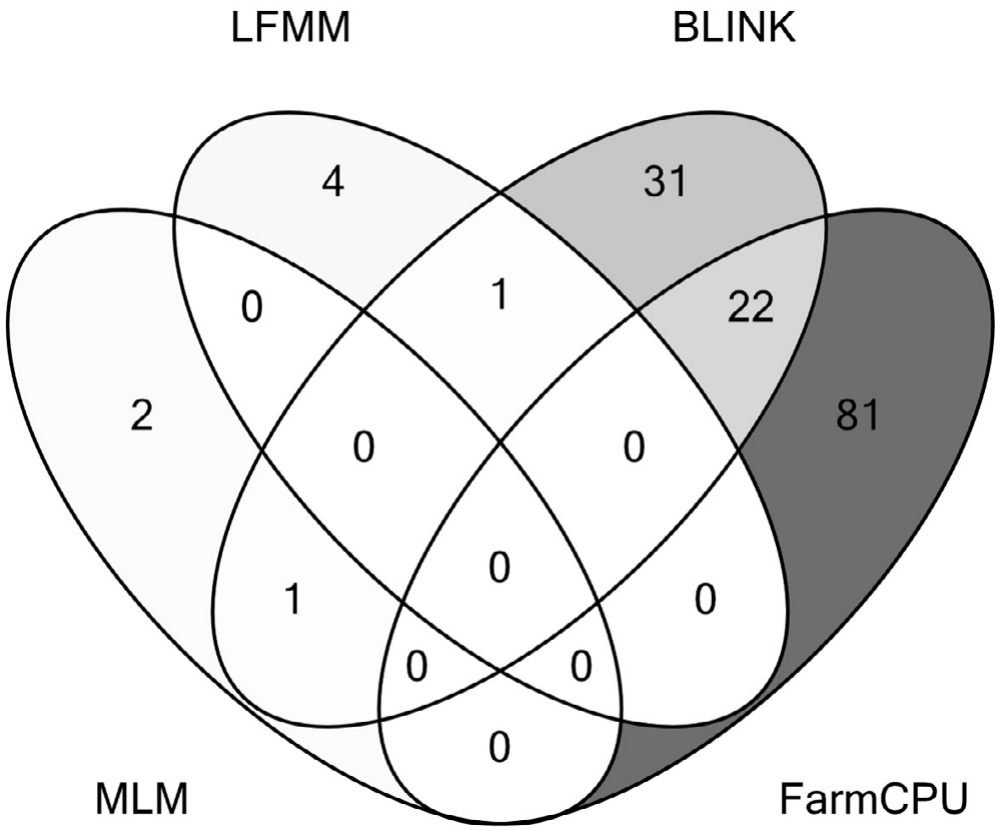
Number of FDR significant environmentally associated SNPs across four models.

To compare the results from each method, the overlap of significant SNPs was analysed (Table S4). One of three significant SNPs from the MLM was detected by BLINK and one of the five LFMM significant SNPs was also significant when using BLINK. A total of 22 significant SNPs overlapped between BLINK and FarmCPU with 31 and 81 SNPs unique to BLINK and FarmCPU, respectively. There was no overlap between MLM, LFMM or FarmCPU and therefore no SNPs were common between all methods (Figure 9).

The function of genes near overlapping significant SNPs was investigated to determine whether any were previously associated with environmental factors; however, they remained as putative targets of selection. For the marker detected in both LFMM and BLINK associated with mean diurnal temperature (BIO2), three genes are found around this locus: *RCS3* (LOC_Os03g53650), *YTH5* (LOC_Os03g53670) and *MyoXIE* (LOC_Os03g53660). These encode a cysteine synthase known to contribute to arsenic tolerance (Wang et al. 2020), a protein shown to influence rice growth, putatively via phytohormone systems (Cai et al. 2023) and a myosin head domain‐containing protein. The other region detected by LFMM (chromosome 4) maps to a glutamine synthase (GS2; LOC_Os04g56400) that confers improved drought and salinity tolerance when co‐overexpressed with *GS1;1* (James et al. 2018).

The region detected using MLM and BLINK mapped to a region in chromosome 8 has four expressed proteins (according to the GenBank name) and one F‐box ubiquitin ligase (LOC_Os08g06710) within 20 bk, although they have no previous links to abiotic stresses or known molecular functions.

The 22 significant markers detected by both BLINK and FarmCPU are distributed across all but chromosomes 5 and 8 (Table S4). In chromosome 1, *SE13* (LOC_Os01g72090) was identified near a marker associated with BIO14 (precipitation of the driest month) and BIO19 (precipitation of the coldest quarter). This gene encodes a phytochromobilin synthase involved in phytochrome biosynthesis (Reig‐Valiente et al. 2020) and therefore potentially regulates light reception and flowering time, a trait known to vary across the range of *O. sativa* (Shrestha et al. 2014). A significant SNP in chromosome 2, associated with mean annual temperature, is close to a gene encoding a receptor‐like cytoplasmic protein kinase (RLCPK72; LOC_Os02g30900) that is upregulated during cold, drought and salt stresses (Vij et al. 2008). SNPs close to the genes *ALS1* (LOC_Os03g54790) involved in aluminium detoxification (Zhu et al. 2018) and *MORF9* (LOC_Os08g04450) differentially expressed based on temperature (Zhang et al. 2019) were associated with BIO1. A SNP in proximity to *RSUS4* (LOC_Os03g22120), induced by salinity treatment (Zhang et al. 2022), was associated with BIO18 (precipitation of the warmest quarter) and a SNP in chromosome 10 was associated with BIO8 (temperature of the wettest quarter) near to genes *HAG702*, differentially upregulated in stress (Hou et al. 2021) and *ONI2*, involved in cuticular wax synthesis (Tsuda et al. 2012) and also differentially regulated by stress (Gao et al. 2022). Finally, a SNP in proximity to *XA21* (LOC_Os11g35500), which is well known to function in resistance to *Xanthomonas oryzae* (Century et al. 1999) but may also provide improved drought tolerance (Shamsunnaher et al. 2020) was associated with BIO19.

## 4 Discussion

We compared 138 studies which had used EAA approaches to associate genetic markers and/or genomic loci to climatic variables. Overall, there was little overlap in the approaches used, with a range of methods, cut‐offs and statistics employed.

Herbaceous (45%) and woody species (55%) were researched in proportions similar to the life history of plants on Earth (Fitzjohn et al. 2014), but present different qualities. Woody species may be easier to sample in terms of locally adapted material and accuracy of bioclimatic data but may be more challenging to investigate in reciprocal transplant experiments compared to annual herbaceous species (Leimu and Fischer 2008). There was a general split in the literature based on whether studies aimed to understand the processes of local adaptation, e.g., to inform conservation, or to identify putative locally adapted genes for crop improvement, often for future climates. For example, Rellstab et al. (2016) used EAA alongside risk of non‐adaptedness with the aim of generating data to inform guidelines for future management of oak species, with different climate factors displaying varying importance across species. In contrast, other studies aim to identify alleles associated with environmental adaptation for the purposes of crop breeding (e.g., Lei et al. 2019; Li et al. 2020).

We showed that targeting candidate genes to identify markers and that pre‐filtering based on some measure of differentiation resulted in a different proportion of significant market associations. Both results are expected as in each case the method enriches for potential EA markers based on prior knowledge (e.g., flowering time genes) or differentiation between populations in different environments (e.g., *F*_ST_). These methods provide a targeted approach for EA marker detection; however, detection of EA markers is more restricted compared to an analysis using a set of genome‐wide markers with no pre‐filtering and will likely miss associations.

Many factors varied between studies, including the target species, number of accessions and markers, statistical method, filtering parameters and significance criteria. It is therefore unlikely that any strong trends between a single factor and ability to detect significant markers would be identified. For example, we found no correlation between missing data threshold and the number of associated SNPs, which was also shown previously (Ahrens et al. 2018). Despite this, simulated changes in MAF and missing data thresholds predict that relaxed thresholds result in more true positives at the expense of more false positives when using BayEnv2 and LFMM (Ahrens et al. 2021). Additionally, the accuracy of climate data may factor in the detection of EA markers. For instance, the widely used climate dataset WorldClim consists of averages of 1970–2000 data interpolated at four resolutions ranging from ~1 km^2^ to ~18 km^2^ grids (Fick and Hijmans 2017). Therefore, there may be spatial and temporal variation based on when and where individuals were sampled from. Overall, the large degree of methodological variation makes it challenging to compare the results of EAA studies at present.

We also showed that many studies carried out GO enrichment of candidate genes but found no enriched terms. This could be due to (1) linkage of non‐causative genes to the actual target of selection, and/or (2) the large diversity in climate variables often analysed, i.e., multiple unrelated biological processes may be associated with different climate variables meaning no global enrichment is identified.

To provide a controlled comparison of different approaches we applied four widely used approaches and consistent cut‐offs to a dataset of rice landraces from Vietnam. We found little evidence of outlier clustering in the rice genome; however, this could be a result of the low SNP density, with an average and median of 5.74 and 5 SNPs per 100 kb, respectively, or inflation of false positive results. There was limited overlap between the four methods, which is expected as previous studies have reported considerable variation in the significant associations detected, even when analysing the same genetic and environmental data. As a clear example of this, Frichot et al. (2013) reanalysed data from Eckert et al. (2010) using LFMM instead of a GLM analysis, finding 113 significant SNPs associated with environmental data compared to a previous 5 significant associations. Chang et al. (2022) identified 352, 279, 307, 364 SNPs associated with the environment in wild barley using a simple RDA, BAYPASS, LFMM and partial RDA methods, respectively, with only 186 of 1107 SNPs overlapping between two or more methods. Also in barley, Chen et al. (2023) detected 92 SNPs using LFMM and 45 with FarmCPU with only two SNPs common between models and Abebe et al. (2015) found 18 and 62 environmentally associated loci with BayEnv and LFMM, respectively, but with no overlap between approaches. Collectively, these findings highlight that if a study relies on only one method to identify associations (which was substantially more common than using multiple approaches in our literature survey), there should be justification that this method is the most appropriate based on details of the data and samples being analysed (elaborated below).

## 5 Conclusions and Future Directions

Overall, our literature review reveals the broad range of taxa studied in EAAs, with studies generally aiming to detect candidate EA genes for crop breeding, to understand local adaptation or to inform conservation. It highlights the large variation in the number of accessions and markers used, statistical methods selected, marker filtering parameters, significance thresholds and methods of candidate marker/gene selection. This inconsistency results in an inability to directly compare the results from these studies or to fully analyse the results. Our EAA of Vietnamese rice confirms the disparity in the number and identity of associated markers between four common EAA models, further emphasising the importance of reporting methodological decisions. A summary of the frequently used EAA models is presented in Table 1.

**TABLE 1:** Summary of three frequently selected EAA models, detailing the statistical implementation, strengths and weaknesses of the models.

Method	Implementation	Strengths	Weaknesses	References
MLM	A mixed linear model incorporating environmental variables as fixed effects and modelling of population relatedness (kinship).	Controls for population structure High power to detect true positives	Computationally intensive Prone to high false positive rates if population structure is not adequately controlled	Rellstab et al. (2015), Yoder and Tiffin (2017)
LFMM	A mixed model incorporating environmental variables as fixed effects and population structure as random (latent) factors (K value)	Controls for population structure Low false negative and false positive rates Less computationally intensive	Requires the K‐value to be predetermined Lower power to detect alleles displaying conditional neutrality	Frichot et al. (2013), Rellstab et al. (2015), Yoder and Tiffin (2017)
BayEnv	A Bayesian method based on allele frequencies. Tests whether a model including environmental variables provides a better fit than one without, at each locus.	Controls for population structure Designed specifically for EAAs Can analyse pooled sequence data (BayEnv2)	Computationally intensive High run‐to‐run variability Lower detection power	Günther and Coop (2013), Blair et al. (2014), Rellstab et al. (2015), Yoder and Tiffin (2017)

Our findings highlight that method and approach are key to the results from an EEA. Our data, and those of Frichot et al. (2013), Chang et al. (2022), Chen et al. (2023) and Abebe et al. (2015), show that different outliers are identified with different computational approaches, with limited overlap. While it is tempting to suggest that only those evident in more than one analysis should be considered true positives, it must be remembered that the models are making different assumptions about the data, some of which might be relevant to a specific study design. For example, kinship is considered in the MLM approach and the genetic architecture of the traits (number of QTL and variance explained by each QTL) will affect performance of the models differently (Merrick et al. 2022). The relative importance of outliers resolved from each method should be judged considering assumptions of the model used to identify them. If selection of candidate SNPs is done by comparing the outputs from several models, this should be done in a way that accounts for linkage disequilibrium. Models such as BLINK will iteratively incorporate covarying SNPs into the model; therefore, different SNPs may associate with the same QTL across models.

Further work should therefore aim to establish a set of best practices to aid future EAA studies. Benchmarking pipelines should be applied to simulated and existing datasets, such as those identified in Campbell et al. (2025). Processing genotype data based on combinations of several factors will enable the testing of dataset suitability and the impact of each methodological decision in the EAA pipeline. Examples of such criteria include the number of georeferenced accessions, the number of SNPs, minor allele frequency and missing data thresholds, levels of SNP linkage disequilibrium pruning, EAA model choice and the significance thresholds selected. Statistical techniques frequently applied to GWAS analyses, such as power analysis, will aid in determining the suitability of the datasets. Further, it could also be beneficial to incorporate a variety of genotype data qualities to determine whether the best practices require adjusting for lower quality datasets, for example for low SNP density and low coverage genotyping.

In addition to using existing datasets, it is likely that simulating data with the approach employed by Lotterhos and Whitlock (2015), will enable the testing of EAA methodologies by revealing the rates of true and false positive marker detections. This could be achieved similar to previous studies that have revealed that the number of accessions (Lotterhos and Whitlock 2015), species breeding strategy (De Mita et al. 2013) and sampling strategy, such as gradient sampling versus clustered populations (De Mita et al. 2013; Yoder and Tiffin 2017), can all have an impact on the detection of environmentally associated markers.

The literature search also revealed that the results from EAA studies in crops are, with very few exceptions, not being validated or applied to crop breeding, despite this being the broad context for most of these studies. Therefore, despite the *potential* of EAAs in detecting accessions, markers and alleles putatively adapted to various environments, our analysis of literature reveals that the results from EAA studies are not yet being effectively utilised.

Echoing a previous review, we suggest the implementation of the GO term ‘Environmentally Associated’ to improve the accessibility of EAA results (Waldvogel et al. 2020), although this should only be applied to validated associations or strong candidate genes due to the tendency of false positives. Experimental validation would be ideal, and may involve the use of reciprocal transplants, using the EA SNPs to predict fitness changes (Rellstab et al. 2015), providing strong support for local adaptation. Alternatively, validation may be achieved through mutagenesis approaches, similar to validation of GWAS candidates (e.g., Curtin et al. 2017), however, this may be restricted to associations where the environmental variable can be experimentally replicated and reliably tested, e.g., when not affected by highly correlated environmental variables.

Without experimental validation, overlap with data from other sources could be sufficient. A strong candidate may have known functional information that is related to the associated environmental factor, such as drought tolerance associated with low precipitation or high temperature variables. Alternatively, or in addition, markers or genes identified across multiple independent populations associated with similar environmental factors are also stronger candidates, similar to meta‐GWAS approaches (Albert and Sauvage 2022). Candidate genes overlapping with those identified through complementary approaches are also suitable candidates, for example if there were overlap with genes identified in a transcriptome‐wide association study (TWAS) as transcript expression is largely (but not always) independent of LD (Albert and Sauvage 2022).

We recommend that reporting standards are employed for all methodological steps, and for reporting associations, especially including gene model IDs to aid searching and cross‐referencing across databases of EA candidate genes. Previously, reporting standards have been proposed and employed in other fields, for example microarray analysis (Brazma et al. 2001) and more recently considerations have been made for GWAS (Uffelmann et al. 2021) to ensure transparency and reproducibility, and we hope the EAA field can consider the same approach. Finally, collaborative efforts between bioinformatic and molecular groups are likely to improve and streamline the validation of EAA candidate genes. Together these suggestions may aid the application of EAA results, for example for crop improvement.

## Author Contributions

All authors designed research, J.A.B. performed research, analysed data and wrote the first version of the manuscript. All authors read, edited, and approved the final version.

## Funding

This work was supported by the Natural Environmental Research Council for the INSPIRE DTP at the University of Southampton (grant number NE/S007210/1).

## Ethics Statement

No ethical considerations are relevant to this submission, no fresh samples were collected or used in this work; instead existing data was used with all relevant data sources referenced. All code and data used is publicly available. We follow the Committee on Publication Ethics (COPE) including not using AI or LLMs at any point during this work.

## Consent

The authors have nothing to report.

## Conflicts of Interest

The authors declare no conflicts of interest.

## Supporting information


**Figure S1:** Visual outputs from the association analysis of bioclimatic variables across four models. Manhattan plots illustrate the distribution of markers across the genome and the significance of their association on a ‐log_10_(*p*) scale. Coloured points represent markers passing false discovery and the dashed line is the Bonferroni threshold (*α* = 0.05). The quantile‐quantile plots display the observed *p* values for each model against the expected *p* values for a null association (as indicated by the red line).

**Table S1:** Details of environmental association analysis publications, including information on the species analysed, number of accessions, EAA method, genetic marker details and the number of significant environmentally associated markers detected. MAF, Minor allele frequency; MD, Missing data; *R*, Random; S, Selective.
**Table S2:** Bioclimatic and elevation variable keys and descriptions. Obtained from WorldClim (Hijmans et al. 2005) and SRTM 90‐m resolution data sets.
**Table S3:** Genetic markers passing the false discorvery rate (FDR) threshold in association with bioclimating variables across the four models analysed.
**Table S4:** Markers detected across multiple models and the predicted genes present within 10 kb of the significant marker.

## Data Availability

All code and data used is publicly available.
