# Supplementary material for: Environmental Association Analyses in Plants and the Influence of Methodological Decisions
Source: Mol Ecol. 2026 Aug 13;35(16):e70508. doi: 10.1111/mec.70508 (PMC13469997; doi:10.1111/mec.70508)
Supplement: Supplementary file 1 [file MEC-35-e70508-s001.docx]

**Supplementary Figure 1 –** Visual outputs from the association analysis of bioclimatic variables across four models. Manhattan plots illustrate the distribution of markers across the genome and the significance of their association on a -log_10_(*p*) scale. Coloured points represent markers passing false discovery and the dashed line is the Bonferroni threshold (α = 0.05). The quantile-quantile plots display the observed *p* values for each model against the expected *p* values for a null association (as indicated by the red line).
