# Supplementary material for: Environmental Association Analyses in Plants and the Influence of Methodological Decisions
Source: Mol Ecol. 2026 Aug 13;35(16):e70508. doi: 10.1111/mec.70508 (PMC13469997; doi:10.1111/mec.70508)
Supplement: Supplementary file 2 [file MEC-35-e70508-s002.zip › mec70508-references.docx]

References

Aguirre-Liguori, J. A., Morales-Cruz, A. & Gaut, B. S. (2022). Evaluating the Persistence and Utility of Five Wild Vitis Species in the Context of Climate Change. Molecular Ecology, 31, 6457–6472. doi:10.1111/mec.16715

Anderson, J. E., Kono, T. J. Y., Stupar, R. M., Kantar, M. B. & Morrell, P. L. (2016). Environmental Association Analyses Identify Candidates for Abiotic Stress Tolerance in Glycine soja, the Wild Progenitor of Cultivated Soybeans. G3: Genes|Genomes|Genetics, 6, 835–843. doi:10.1534/g3.116.026914

Andrello, M., Henry, K., Devaux, P., Desprez, B. & Manel, S. (2016). Taxonomic, Spatial and Adaptive Genetic Variation of Beta Section Beta. Theoretical and Applied Genetics, 129, 257–271. doi:10.1007/s00122-015-2625-7

Bashalkhanov, S., Eckert, A. J. & Rajora, O. P. (2013). Genetic Signatures of Natural Selection in Response to Air Pollution in Red Spruce (Picea rubens, Pinaceae). Molecular Ecology, 22, 5877–5889. doi:10.1111/mec.12546

Berny Mier Y Teran, J. C., Konzen, E. R., Medina, V., Palkovic, A., Ariani, A., Tsai, S. M., Gilbert M. E., Gepts P. (2018). Root and Shoot Variation in Relation to Potential Intermittent Drought Adaptation of Mesoamerican Wild Common Bean (Phaseolus vulgaris L.). Annals of Botany, 124, 917–932. doi:10.1093/aob/mcy221

Blanco-Pastor, J. L., Barre, P., Keep, T., Ledauphin, T., Escobar-Gutiérrez, A., Roschanski, A. M., Willner E., Dehmer K. J., Hegarty M., Muylle H., Veeckman E., Vandepoele K., Ruttink T., Roldán-Ruiz I., Manel S., Sampoux J.P. (2021a). Canonical Correlations Reveal Adaptive Loci and Phenotypic Responses to Climate in Perennial Ryegrass. Molecular Ecology Resources, 21, 849–870. doi:10.1111/1755-0998.13289

Blanco-Pastor, J. L., Liberal, I. M., Sakiroglu, M., Wei, Y., Brummer, E. C., Andrew, R. L., Pfeil B. E. (2021b). Annual and Perennial Medicago Show Signatures of Parallel Adaptation to Climate and Soil in Highly Conserved Genes. Molecular Ecology, 30, 4448–4465. doi:10.1111/mec.16061

Blois, L., De miguel, M., Bert, P. F., Girollet, N., Ollat, N., Rubio, B., et al. (2023). Genetic Structure and First Genome-Wide Insights Into the Adaptation of a Wild Relative of Grapevine, Vitis berlandieri. Evolutionary Applications, 16, 1184–1200. doi:10.1111/eva.13566

Borrell, J. S., Zohren, J., Nichols, R. A. & Buggs, R. J. A. (2020). Genomic Assessment of Local Adaptation in Dwarf Birch to Inform Assisted Gene Flow. Evolutionary Applications, 13, 161–175. doi:10.1111/eva.12883

Brunazzi, A., Scaglione, D., Talini, R. F., Miculan, M., Magni, F., Poland, J., Enrico Pè M., Brandolini A., Dell'Acqua M. (2018). Molecular Diversity and Landscape Genomics of the Crop Wild Relative Triticum urartu Across the Fertile Crescent. The Plant Journal, 94, 670–684. doi:10.1111/tpj.13888

Caproni, L., Lakew, B. F., Kassaw, S. A., Miculan, M., Ahmed, J. S., Grazioli, S., Kidane Y. G., Fadda C., Pè M. E., Dell'Acqua M. (2023). The Genomic and Bioclimatic Characterization of Ethiopian Barley (Hordeum vulgare L.) Unveils Challenges and Opportunities to Adapt to a Changing Climate. Global Change Biology, 29, 2335–2350. doi:10.1111/gcb.16560

Chen, J., Källman, T., Ma, X., Gyllenstrand, N., Zaina, G., Morgante, M., Bousquet J., Eckert A., Wegrzyn J., Neale D., Lagercrantz U., Lascoux M. (2012). Disentangling the Roles of History and Local Selection in Shaping Clinal Variation of Allele Frequencies and Gene Expression in Norway Spruce (Picea abies). Genetics, 191, 865–881. doi:10.1534/genetics.112.140749

Cortés, A. J. & Blair, M. W. (2018). Genotyping by Sequencing and Genome–Environment Associations in Wild Common Bean Predict Widespread Divergent Adaptation to Drought. Frontiers in Plant Science, 9. doi:10.3389/fpls.2018.00128, 128

Dell'acqua, M., Zuccolo, A., Tuna, M., Gianfranceschi, L. & Pè, M. E. (2014). Targeting Environmental Adaptation in the Monocot Model Brachypodium distachyon: A Multi-Faceted Approach. BMC Genomics, 15, 801. doi:10.1186/1471-2164-15-801

Eckert, A. J., Maloney, P. E., Vogler, D. R., Jensen, C. E., Mix, A. D. & Neale, D. B. (2015). Local Adaptation at Fine Spatial Scales: An Example From Sugar Pine (Pinus lambertiana, Pinaceae). Tree Genetics & Genomes, 11, 42. doi:10.1007/s11295-015-0863-0

Elias, J. C. F., Gonçalves-Vidigal, M. C., Ariani, A., Valentini, G., Martiniano-Souza, M. D. C., Vaz Bisneta, M., Gepts P. (2021). Genome-Environment Association Analysis for Bio-Climatic Variables in Common Bean (Phaseolus vulgaris L.) From Brazil. Plants, 10, 1572. doi:10.3390/plants10081572

Evans, L. M., Slavov, G. T., Rodgers-Melnick, E., Martin, J., Ranjan, P., Muchero, W., Brunner A. M., Schackwitz W., Gunter L., Chen J.G., Tuskan G. A., DiFazio S. P. (2014). Population Genomics of Populus trichocarpa Identifies Signatures of Selection and Adaptive Trait Associations. Nature Genetics, 46, 1089–1096. doi:10.1038/ng.3075

Fahrenkrog, A. M., Neves, L. G., Resende Jr, M. F. R., Dervinis, C., Davenport, R., Barbazuk, W. B., Kirst M. (2017). Population Genomics of the Eastern Cottonwood (Populus deltoides). Ecology and Evolution, 7, 9426–9440. doi:10.1002/ece3.3466

Fang, Z., Gonzales, A. M., Clegg, M. T., Smith, K. P., Muehlbauer, G. J., Steffenson, B. J., Morrell P. L. (2014). Two Genomic Regions Contribute Disproportionately to Geographic Differentiation in Wild Barley. G3: Genes|Genomes|Genetics, 4, 1193–1203. doi:10.1534/g3.114.010561

Faye, J. M., Maina, F., Hu, Z., Fonceka, D., Cisse, N. & Morris, G. P. (2019). Genomic Signatures of Adaptation to Sahelian and Soudanian Climates in Sorghum Landraces of Senegal. Ecology and Evolution, 9, 6038–6051. doi:10.1002/ece3.5187

Feng, S., Xi, E., Wan, W. & Ru, D. (2023). Genomic Signals of Local Adaptation in Picea Crassifolia. BMC Plant Biology, 23, 534. doi:10.1186/s12870-023-04539-7

Fischer, M. C., Rellstab, C., Tedder, A., Zoller, S., Gugerli, F., Shimizu, K. K., Holderegger R., Widmer A. (2013). Population Genomic Footprints of Selection and Associations With Climate in Natural Populations of Arabidopsis Halleri From the Alps. Molecular Ecology, 22, 5594–5607. doi:10.1111/mec.12521

Fitzpatrick, M. C., Chhatre, V. E., Soolanayakanahally, R. Y. & Keller, S. R. (2021). Experimental Support for Genomic Prediction of Climate Maladaptation Using the Machine Learning Approach Gradient Forests. Molecular Ecology Resources, 21, 2749–2765. doi:10.1111/1755-0998.13374

Fournier-Level, A., Korte, A., Cooper, M. D., Nordborg, M., Schmitt, J. & Wilczek, A. M. (2011). A Map of Local Adaptation in Arabidopsis thaliana. Science, 334, 86–89. doi:10.1126/science.1209271

Fufa, T. W., Menamo, T. M., Abtew, W. G., Amadi, C. O. & Oselebe, H. O. (2023). Detection of the Local Adaptive and Genome-Wide Associated Loci in Southeast Nigerian Taro (Colocasia esculenta (L.) Schott) Populations. BMC Genomics, 24, 39. doi:10.1186/s12864-023-09134-6

Geraldes, A., Farzaneh, N., Grassa, C. J., Mckown, A. D., Guy, R. D., Mansfield, S. D., Douglas C. J., Cronk Q. C. B. (2014). Landscape Genomics of Populus trichocarpa: The Role of Hybridization, Limited Gene Flow, and Natural Selection in Shaping Patterns of Population Structure. Evolution, 68, 3260–3280. doi:10.1111/evo.12497

Gibson, M. J. S. & Moyle, L. C. (2020). Regional Differences in the Abiotic Environment Contribute to Genomic Divergence Within a Wild Tomato Species. Molecular Ecology, 29, 2204–2217. doi:10.1111/mec.15477

Girma, G., Nida, H., Tirfessa, A., Lule, D., Bejiga, T., Seyoum, A., Mekonen M., Nega A., Dessalegn K., Birhanu C., Bekele A., Gebreyohannes A., Ayana G., Tesso T., Ejeta G., Mengiste T. (2020). A Comprehensive Phenotypic and Genomic Characterization of Ethiopian Sorghum Germplasm Defines Core Collection and Reveals Rich Genetic Potential in Adaptive Traits. The Plant Genome, 13, e20055. doi:10.1002/tpg2.20055

Godbout, J., Gros-Louis, M.-C., Lamothe, M. & Isabel, N. (2020). Going With the Flow: Intraspecific Variation May Act as a Natural Ally to Counterbalance the Impacts of Global Change for the Riparian Species Populus deltoides. Evolutionary Applications, 13, 176–194. doi:10.1111/eva.12854

Gómez-Espejo, A. L., Sansaloni, C. P., Burgueño, J., Toledo, F. H., Benavides-Mendoza, A. & Reyes-Valdés, M. H. (2022). Worldwide Selection Footprints for Drought and Heat in Bread Wheat (Triticum aestivum L.). Plants, 11, 2289. doi:10.3390/plants11172289

Hancock, A. M., Brachi, B., Faure, N., Horton, M. W., Jarymowycz, L. B., Sperone, F. G., Toomajian C., Roux F., Bergelson J. (2011). Adaptation to Climate Across the Arabidopsis thaliana Genome. Science, 334, 83–86. doi:10.1126/science.1209244

Hanif, U., Alipour, H., Gul, A., Jing, L., Darvishzadeh, R., Amir, R., Munir F., Ilyas M. K., Ghafoor A., Siddiqui S. U., St. Amand P., Bernado A., Bai G., Sonder K., Rasheed A., He Z., Li H. (2021). Characterization of the Genetic Basis of Local Adaptation of Wheat Landraces From Iran and Pakistan Using Genome-Wide Association Study. The Plant Genome, 14, e20096. doi:10.1002/tpg2.20096

He, S., Li, G., Zhang, J., Ding, Y., Wu, H., Xie, J., Wu H., Yang Z. (2023). The Effect of Environmental Factors on the Genetic Differentiation of Cucurbita ficifolia Populations Based on Whole-Genome Resequencing. BMC Plant Biology, 23, 647. doi:10.1186/s12870-023-04602-3

Hu, Y., Feng, C., Yang, L., Edger, P. P. & Kang, M. (2022). Genomic Population Structure and Local Adaptation of the Wild Strawberry Fragaria nilgerrensis. Horticulture Research, 9. doi:10.1093/hr/uhab059, uhab059

Ingvarsson, P. K. & Bernhardsson, C. (2020). Genome-Wide Signatures of Environmental Adaptation in European Aspen (Populus tremula) Under Current and Future Climate Conditions. Evolutionary Applications, 13, 132–142. doi:10.1111/eva.12792

Jones, M. R., Forester, B. R., Teufel, A. I., Adams, R. V., Anstett, D. N., Goodrich, B. A., Landguth E. L., Joost S., Manel S. (2013). Integrating Landscape Genomics and Spatially Explicit Approaches to Detect Loci Under Selection in Clinal Populations. Evolution, 67, 3455–3468. doi:10.1111/evo.12237

Jump, A. S., Hunt, J. M., Martínez-Izquierdo, J. A. & Peñuelas, J. (2006). Natural Selection and Climate Change: Temperature-Linked Spatial and Temporal Trends in Gene Frequency in Fagus sylvatica. Molecular Ecology, 15, 3469–3480. doi:10.1111/j.1365-294X.2006.03027.x

Jump, A. S., Peñuelas, J., Rico, L., Ramallo, E., Estiarte, M., Martínez-Izquierdo , J. A., et al. (2008). Simulated Climate Change Provokes Rapid Genetic Change in the Mediterranean Shrub Fumana Thymifolia. Global Change Biology, 14, 637–643. doi:10.1111/j.1365-2486.2007.01521.x

Keller, S. R., Levsen, N., Olson, M. S. & Tiffin, P. (2012). Local Adaptation in the Flowering-Time Gene Network of Balsam Poplar, Populus balsamifera L. Molecular Biology and Evolution, 29, 3143–3152. doi:10.1093/molbev/mss121

Li, J.-X., Zhu, X.-H., Li, Y., Liu, Y., Qian, Z.-H., Zhang, X.-X., Sun Y., Ji L.Y. (2018). Adaptive Genetic Differentiation in Pterocarya stenoptera (Juglandaceae) Driven by Multiple Environmental Variables Were Revealed by Landscape Genomics. BMC Plant Biology, 18, 306. doi:10.1186/s12870-018-1524-x

Li, Y., Cao, K., Li, N., Zhu, G., Fang, W., Chen, C., Wang X., Guo J., Wang Q., Ding T., Wang J., Guan L., Wang J., Liu K., Guo W., Arús P., Huang S., Fei Z., Wang L. (2021). Genomic Analyses Provide Insights Into Peach Local Adaptation and Responses to Climate Change. Genome Research, 31, 592–606. doi:10.1101/gr.261032.120

López-Hernández, F. & Cortés, A. J. (2019). Last-Generation Genome–Environment Associations Reveal the Genetic Basis of Heat Tolerance in Common Bean (Phaseolus vulgaris L.). Frontiers in Genetics, 10. doi:10.3389/fgene.2019.00954, 954

Manel, S., Gugerli, F., Thuiller, W., Alvarez, N., Legendre, P., Holderegger, R., Holderegger R., Gielly L., Taberlet P. (2012). Broad-Scale Adaptive Genetic Variation in Alpine Plants Is Driven by Temperature and Precipitation. Molecular Ecology, 21, 3729–3738. doi:10.1111/j.1365-294X.2012.05656.x

Manel, S., Poncet, B. N., Legendre, P., Gugerli, F. & Holderegger, R. (2010). Common Factors Drive Adaptive Genetic Variation at Different Spatial Scales in Arabis alpina. Molecular Ecology, 19, 3824–3835. doi:10.1111/j.1365-294X.2010.04716.x

Mariac, C., Jehin, L., Saïdou, A.-A., Thuillet, A.-C., Couderc, M., Sire, P., Jugdé H., Adam H., Bezançon G., Pham J.L., Vigouroux Y. (2011). Genetic Basis of Pearl Millet Adaptation Along an Environmental Gradient Investigated by a Combination of Genome Scan and Association Mapping. Molecular Ecology, 20, 80–91. doi:10.1111/j.1365-294X.2010.04893.x

Martins, K., Gugger, P. F., Llanderal-Mendoza, J., González-Rodríguez, A., Fitz-Gibbon, S. T., Zhao, J.-L., Rodríguez-Correa H., Oyama K., Sork V. L. (2018). Landscape Genomics Provides Evidence of Climate-Associated Genetic Variation in Mexican Populations of Quercus rugosa. Evolutionary Applications, 11, 1842–1858. doi:10.1111/eva.12684

Menamo, T., Kassahun, B., Borrell, A. K., Jordan, D. R., Tao, Y., Hunt, C., Mace E. (2021). Genetic Diversity of Ethiopian Sorghum Reveals Signatures of Climatic Adaptation. Theoretical and Applied Genetics, 134, 731–742. doi:10.1007/s00122-020-03727-5

Miao, C.-Y., Li, Y., Yang, J. & Mao, R.-L. (2017). Landscape Genomics Reveal That Ecological Character Determines Adaptation: A Case Study in Smoke Tree (Cotinus coggygria Scop.). BMC Evolutionary Biology, 17, 202. doi:10.1186/s12862-017-1055-3

Nadeau, S., Meirmans, P. G., Aitken, S. N., Ritland, K. & Isabel, N. (2016). The Challenge of Separating Signatures of Local Adaptation From Those of Isolation by Distance and Colonization History: The Case of Two White Pines. Ecology and Evolution, 6, 8649–8664. doi:10.1002/ece3.2550

Neyhart, J. L., Kantar, M. B., Zalapa, J. & Vorsa, N. (2022). Genomic-Environmental Associations in Wild Cranberry (Vaccinium macrocarpon Ait.). G3: Genes|Genomes|Genetics, 12. doi:10.1093/g3journal/jkac203

Olatoye, M. O., Hu, Z., Maina, F. & Morris, G. P. (2018). Genomic Signatures of Adaptation to a Precipitation Gradient in Nigerian Sorghum. G3: Genes|Genomes|Genetics, 8, 3269–3281. doi:10.1534/g3.118.200551

Poncet, B. N., Herrmann, D., Gugerli, F., Taberlet, P., Holderegger, R., Gielly, L., et al. (2010). Tracking Genes of Ecological Relevance Using a Genome Scan in Two Independent Regional Population Samples of Arabis alpina. Molecular Ecology, 19, 2896–2907. doi:10.1111/j.1365-294X.2010.04696.x

Pournosrat, R., Kaya, S., Shaaf, S., Kilian, B. & Ozkan, H. (2018). Geographical and Environmental Determinants of the Genetic Structure of Wild Barley in Southeastern Anatolia. PLoS One, 13, e0192386. doi:10.1371/journal.pone.0192386

Prunier, J., Laroche, J., Beaulieu, J. & Bousquet, J. (2011). Scanning the Genome for Gene SNPs Related to Climate Adaptation and Estimating Selection at the Molecular Level in Boreal Black Spruce. Molecular Ecology, 20, 1702–1716. doi:10.1111/j.1365-294X.2011.05045.x

Pyhäjärvi, T., Hufford, M. B., Mezmouk, S. & Ross-Ibarra, J. (2013). Complex Patterns of Local Adaptation in Teosinte. Genome Biology and Evolution, 5, 1594–1609. doi:10.1093/gbe/evt109

Rellstab, C., Fischer, M. C., Zoller, S., Graf, R., Tedder, A., Shimizu, K. K., Widmer A., Holderegger R., Gugerli F. (2017). Local Adaptation (Mostly) Remains Local: Reassessing Environmental Associations of Climate-Related Candidate SNPs in Arabidopsis Halleri. Heredity, 118, 193–201. doi:10.1038/hdy.2016.82

Rodriguez, M., Scintu, A., Posadinu, C. M., Xu, Y., Nguyen, C. V., Sun, H., Bitocchi E., Bellucci E., Papa R., Fei Z., Giovannoni J. J., Rau D., Attene G. (2020). GWAS Based on RNA-Seq SNPs and High-Throughput Phenotyping Combined With Climatic Data Highlights the Reservoir of Valuable Genetic Diversity in Regional Tomato Landraces. Genes, 11, 1387. doi:10.3390/genes11111387

Roschanski, A. M., Csilléry, K., Liepelt, S., Oddou-Muratorio, S., Ziegenhagen, B., Huard, F., Ullrich K. K., Postolache D., Vendramin G. G., Fady B. (2016). Evidence of Divergent Selection for Drought and Cold Tolerance at Landscape and Local Scales in Abies alba Mill. In the French Mediterranean Alps. Molecular Ecology, 25, 776–794. doi:10.1111/mec.13516

Russell, J., Mascher, M., Dawson, I. K., Kyriakidis, S., Calixto, C., Freund, F., Bayer M., Milne I., Marshall-Griffiths T., Heinen S., Hofstad A., Sharma R., Himmelbach A., Knauft M., van Zonneveld M., Brown J. W. S., Schmid K., Kilian B., Muehlbauer G. J., Stein N., Waugh R. (2016). Exome Sequencing of Geographically Diverse Barley Landraces and Wild Relatives Gives Insights Into Environmental Adaptation. Nature Genetics, 48, 1024–1030. doi:10.1038/ng.3612

Shryock, D. F., Havrilla, C. A., Defalco, L. A., Esque, T. C., Custer, N. A. & Wood, T. E. (2015). Landscape Genomics of Sphaeralcea ambigua in the Mojave Desert: A Multivariate, Spatially-Explicit Approach to Guide Ecological Restoration. Conservation Genetics, 16, 1303–1317. doi:10.1007/s10592-015-0741-1

Sokolkova, A., Bulyntsev, S. V., Chang, P. L., Carrasquilla-Garcia, N., Igolkina, A. A., Noujdina, N. V., von Wettberg E., Vishnyakova M. A., Cook D. R., Nuzhdin S. V., Samsonova M. G. (2020). Genomic Analysis of Vavilov's Historic Chickpea Landraces Reveals Footprints of Environmental and Human Selection. International Journal of Molecular Sciences, 21, 3952. doi:10.3390/ijms21113952

Sork, V. L., Squire, K., Gugger, P. F., Steele, S. E., Levy, E. D. & Eckert, A. J. (2016). Landscape Genomic Analysis of Candidate Genes for Climate Adaptation in a California Endemic Oak, Quercus lobata. American Journal of Botany, 103, 33–46. doi:10.3732/ajb.1500162

Steane, D. A., Potts, B. M., Mclean, E., Prober, S. M., Stock, W. D., Vaillancourt, R. E., Byrne M. (2014). Genome-Wide Scans Detect Adaptation to Aridity in a Widespread Forest Tree Species. Molecular Ecology, 23, 2500–2513. doi:10.1111/mec.12751

Turner, T. L., Bourne, E. C., Von Wettberg, E. J., Hu, T. T. & Nuzhdin, S. V. (2010). Population Resequencing Reveals Local Adaptation of Arabidopsis lyrata to Serpentine Soils. Nature Genetics, 42, 260–263. doi:10.1038/ng.515

Turner, T. L., Von Wettberg, E. J. & Nuzhdin, S. V. (2008). Genomic Analysis of Differentiation Between Soil Types Reveals Candidate Genes for Local Adaptation in Arabidopsis lyrata. PLoS One, 3, e3183. doi:10.1371/journal.pone.0003183

Wang, H., Coulman, B., Bai, Y., TarˈAn, B. & Biligetu, B. (2023b). Genetic Diversity and Local Adaption of Alfalfa Populations (Medicago sativa L.) Under Long-Term Grazing. Scientific Reports, 13, 1632. doi:10.1038/s41598-023-28521-3

Wang, T., Wang, Z., Xia, F. & Su, Y. (2016). Local Adaptation to Temperature and Precipitation in Naturally Fragmented Populations of Cephalotaxus oliveri, an Endangered Conifer Endemic to China. Scientific Reports, 6, 25031. doi:10.1038/srep25031

Wang, Y., Zhang, L., Zhou, Y., Ma, W., Li, M., Guo, P., Feng L., Fu C. (2023c). Using Landscape Genomics to Assess Local Adaptation and Genomic Vulnerability of a Perennial Herb Tetrastigma hemsleyanum (Vitaceae) in Subtropical China. Frontiers in Genetics, 14. doi:10.3389/fgene.2023.1150704, 1150704

Westengen, O. T., Berg, P. R., Kent, M. P. & Brysting, A. K. (2012). Spatial Structure and Climatic Adaptation in African Maize Revealed by Surveying SNP Diversity in Relation to Global Breeding and Landrace Panels. PLoS One, 7, e47832. doi:10.1371/journal.pone.0047832

Wu, T., Wen, H., Zhang, X., Jia, H., Xu, C., Song, W., Jiang B., Yuan S., Sun S., Wu C., Han T. (2023). Genome-Wide Association Study for Temperature Response and Photo-Thermal Interaction of Flowering Time in Soybean Using a Panel of Cultivars With Diverse Maturity Groups. Theoretical and Applied Genetics, 136, 245. doi:10.1007/s00122-023-04496-7

Yang, J., Li, Y., Miao, C.-Y. & Mao, R.-L. (2017a). Landscape Genomics Analysis of Achyranthes bidentata Reveal Adaptive Genetic Variations Are Driven by Environmental Variations Relating to Ecological Habit. Population Ecology, 59, 355–362. doi:10.1007/s10144-017-0599-9

Yang, J., Miao, C.-Y., Mao, R.-L. & Li, Y. (2017b). Landscape Population Genomics of Forsythia (Forsythia suspensa) Reveal That Ecological Habitats Determine the Adaptive Evolution of Species. Frontiers in Plant Science, 8. doi:10.3389/fpls.2017.00481, 481

Zhang, X.-X., Liu, B.-G., Li, Y., Liu, Y., He, Y.-X., Qian, Z.-H., Li J.X. (2019b). Landscape Genetics Reveals That Adaptive Genetic Divergence in Pinus bungeana (Pinaceae) is Driven by Environmental Variables Relating to Ecological Habitats. BMC Evolutionary Biology, 19, 160. doi:10.1186/s12862-019-1489-x

Zhang, X., Sun, Y., Landis, J. B., Zhang, J., Yang, L., Lin, N., Zhang H., Guo R., Li L., Zhang Y., Deng T., Sun H., Wang H. (2020). Genomic Insights Into Adaptation to Heterogeneous Environments for the Ancient Relictual Circaeaster Agrestis (Circaeasteraceae, Ranunculales). New Phytologist, 228, 285–301. doi:10.1111/nph.16669

Zulliger, D., Schnyder, E. & Gugerli, F. (2013). Are Adaptive Loci Transferable Across Genomes of Related Species? Outlier and Environmental Association Analyses in Alpine Brassicaceae Species. Molecular Ecology, 22, 1626–1639. doi:10.1111/mec.12199
